# Employing a magnetic chitosan/molybdenum disulfide nanocomposite for efficiently removing polycyclic aromatic hydrocarbons from milk samples

**DOI:** 10.1038/s41598-024-66087-w

**Published:** 2024-07-01

**Authors:** Alieh Rezagholizade-shirvan, Mansoureh Mohammadi, Yeganeh Mazaheri, Saeid Fallahizadeh, Haniyeh Ghorbani, Samira Shokri, Nabi Shariatifar, Majid Darroudi, Ehsan Shamloo

**Affiliations:** 1 Department of Food Science and Technology, Neyshabur University of Medical Sciences, Neyshabur, Iran; 2 Department of Food Science and Technology, Faculty of Nutrition Science and Food Technology, National Nutrition and Food Technology Research Institute, Shahid Beheshti University of Medical Sciences, Tehran, Iran; 3 Food Safety Division, Department of Environmental Health, Faculty of Public Health, Tehran University of Medical Sciences, Tehran, Iran; 4 School of Public Health, Yasuj University of Medical Sciences, Yasuj, Iran; 5 Social Determinants of Health Research Center, Yasuj University of Medical Sciences, Yasuj, Iran; 6 Razi Research Center, Khorasan Razavi Education, Mashhad, Iran; 7 Nuclear Medicine Research Center, Mashhad University of Medical Sciences, Mashhad, Iran

**Keywords:** Adsorption removal, Chitosan, Milk sample, Molybdenum disulfide, Nanocomposite, Polycyclic aromatic hydrocarbons, Environmental sciences, Chemistry

## Abstract

This study aimed to develop a highly efficient nanocomposite composed of magnetic chitosan/molybdenum disulfide (CS/MoS_2_/Fe_3_O_4_) for the removal of three polycyclic aromatic hydrocarbons (PAHs)—pyrene, anthracene, and phenanthrene. Novelty was introduced through the innovative synthesis procedure and the utilization of magnetic properties for enhanced adsorption capabilities. Additionally, the greenness of chitosan as a sorbent component was emphasized, highlighting its biodegradability and low environmental impact compared to traditional sorbents. Factors influencing PAH adsorption, such as nanocomposite dosage, initial PAH concentration, pH, and contact time, were systematically investigated and optimized. The results revealed that optimal removal efficiencies were attained at an initial PAH concentration of 150 mg/L, a sorbent dose of 0.045 g, pH 6.0, and a contact time of 150 min. The pseudo-second-order kinetic model exhibited superior fitting to the experimental data, indicating an equilibrium time of approximately 150 min. Moreover, the equilibrium adsorption process followed the Freundlich isotherm model, with k_f_ and n values exceeding 7.91 mg/g and 1.20, respectively. Remarkably, the maximum absorption capacities for phenanthrene, anthracene, and pyrene on the sorbent were determined as 217 mg/g, 204 mg/g, and 222 mg/g, respectively. These findings underscore the significant potential of the CS/MoS_2_/Fe_3_O_4_ nanocomposite for efficiently removing PAHs from milk and other dairy products, thereby contributing to improved food safety and public health.

## Introduction

Polycyclic aromatic hydrocarbons (PAHs) are characterized by the presence of two or more interconnected aromatic or benzene rings arranged in linear, angular, or cluster structures. They can occur naturally through processes such as forest fires and volcanic eruptions^1,2^, as well as being generated by environmental pollutants, incomplete combustion, and industrial processing^3^. The presence of PAHs is a cause for concern due to their carcinogenic, mutagenic, and toxic properties, as well as their potential for long-range transport and adverse effects on human health^4^. PAHs have been characterized as major pollutants possessing genotoxic characteristics and the potential to cause cancer, as classified by reputable international organizations^5–7^.

The main route of human exposure to PAHs is through food consumption, particularly when consuming contaminated food products. This accounts for more than 80% of total PAH exposure^8,9^. Once ingested, PAHs can become metabolically activated within cells, leading to the formation of diol-epoxides. These diol-epoxides can bind to macromolecules, including DNA, causing disruptions in replication and mutations. Ultimately, this process contributes to the development of a carcinogenic state^10,11^. Milk and dairy products are commonly consumed in human diets, especially by children and the elderly^12,13^. PAHs, being lipophilic, tend to accumulate in the food chain, particularly in high-fat foods^14,15^. Animal-derived products like milk are significant sources of PAH contamination. Ruminant milk, in particular, can contain elevated levels of PAHs due to the consumption of contaminated feed and grass in proximity to industrial areas, as well as the ingestion of contaminated soil. Therefore, effective approaches are urgently needed to reduce the concentration of PAHs, specifically phenanthrene, anthracene, and pyrene, in milk and dairy products.

Adsorptive removal techniques provide numerous advantages for the removal of PAHs in food samples^16–18^. The selective and specific nature of these techniques ensures the targeted removal of PAH compounds, reducing the risk of contamination and protecting public health. Rapid and efficient removal, versatile application, non-toxicity, and cost-effectiveness further enhance the appeal of adsorptive removal methods^19,20^. By incorporating these techniques into milk processing and quality control, food safety can be enhanced, ensuring that milk products are free from PAH contaminants and providing consumers with safe and wholesome dairy products. The success and effectiveness of this technique heavily depend on the choice and performance of the sorbent material^21^. Sorbents play a crucial role in adsorptive removal processes by selectively attracting and retaining target contaminants^22,23^. Recently, nanomaterials and nanocomposites have gained attention as a potential solution for PAH removal^24,25^. The use of these materials as sorbents provides several benefits including versatility, cost reduction, high efficiency in removing contaminants, quick cleanup, and the possibility of regenerating and reusing the sorbent materials.^26,27^. A magnetic chitosan/MoS_2_ nanocomposite, combining the biopolymer chitosan with molybdenum sulfide (MoS_2_) and incorporating magnetic properties through the inclusion of materials like Fe_3_O_4_ nanoparticles, has been developed for various applications, including heavy metal removal and antibacterial activity^28–30^. This nanocomposite exhibits strong magnetic properties and has demonstrated a high adsorption capacity for various compounds from aqueous samples, such as *N,N*-diethylaniline, *N,N*-dimethylaniline, and aniline^31^. In this study, our objective is to synthesize a non-toxic sorbent, namely magnetic chitosan/MoS_2_ nanocomposite, using a simple chemical procedure. The synthesized sorbent is then utilized for the effective removal of carcinogenic PAH compounds, specifically phenanthrene, anthracene, and pyrene, from milk samples. Furthermore, we investigate the isotherm and kinetic of the adsorption process for these three PAHs on the developed sorbent. In this study, our aim is to fabricate a biocompatible and innovative sorbent, the magnetic chitosan/MoS_2_ nanocomposite, employing a straightforward chemical synthesis method. This sorbent is then employed for the efficient removal of carcinogenic PAH compounds, specifically targeting phenanthrene, anthracene, and pyrene, from milk samples. Additionally, we investigated the kinetics of PAH adsorption using the sorbent, elucidating the underlying mechanisms of the process. Furthermore, we examined the equilibrium adsorption behavior of PAHs utilizing both the Freundlich and Langmuir isotherm models, providing insights into the sorption process at equilibrium. Ultimately, the sorbent was employed to extract PAHs from spiked milk samples, serving as representative real food samples with complex matrices. This approach aimed to assess the sorbent's efficacy in removing PAHs from actual food samples under realistic conditions.

## Method and materials

### Materials

Chitosan with a low molecular weight and a degree of deacetylation of 80%, molybdenum trioxide, urea, thioacetamide, and ethanol were obtained from Sigma–Aldrich (St Louis, MO, USA). glutaraldehyde (a 50% solution in distilled water), sodium triphenylphosphine (TPP), glacial acetic acid (98% purity), ammonium hydroxide, manganese chloride tetrahydrate, hydrochloric acid (37% v/v), and sodium hydroxide were purchased from Merck (Darmstadt, Germany). Magnetic Fe_3_O_4_ were purchased from US Research Nanomaterials, Inc. PAH standards, including pyrene (PYR), phenanthrene (PHEN), and anthracene (ANTH), were obtained from Aldrich (Milwaukee, WI, USA). To prepare the standard solution for each PAH, HPLC-grade methanol was utilized to create a stock solution of each PAH (500 mg/L), and raw milk sampling was purchased from markets (Neyshabur, Iran).

### Preparation of MoS_2_ nanoparticles

A precise mixture of urea (8.00 g), MoO_3_ (0.96 g), and thioacetamide (1.123 g) was carefully poured into deionized water (80 mL), ensuring thorough mixing by employing magnetic stirring for 1 h to achieve a uniform solution. Afterward, the solution was moved to an autoclave and exposed to 200 °C for 12 h. After the completion of the heating process, the autoclave was allowed to cool gradually to reach the ambient temperature of 25 °C. The resulting product was then meticulously rinsed with deionized water and ethanol, respectively. Finally, MoS_2_ nanosheets as the product was dried for 12 h at 60 °C to ensure complete removal of moisture^32^.

### Cs/MoS_2_/Fe_3_O_4_ nanocomposite preparation

The synthesis of the hybrid nanocomposite, comprising chitosan and MoS_2_, involved a two-step process. Initially, a chitosan solution with a concentration of 0.2% w/v was prepared by dissolving chitosan (0.1 g) in a CH_3_COOH solution (50 mL, 1% v/v) under magnetic stirring at 25 °C. Then, the MoS_2_ nanosheets were poured into the chitosan solution and subjected to sonicate for 5 h to achieve a MoS_2_-dispersed chitosan matrix with a weight/volume ratio of 0.1:0.2%. The resulting mixture was centrifuged at 6000 rpm for 15 min to separate the solution. In the subsequent step, 20 mg of Fe_3_O_4_ nanoparticles were introduced into the MoS_2_-dispersed chitosan solution and mechanically stirred for 2 h at 25 °C. A combination of 10 mL of paraffin and 4 mL of Span-80 emulsifier was added to the dispersion mixture at 40 °C for over 1 h. After this, a solution of glutaraldehyde (5 mL; 2.5% v/v) was added dropwise into the mixture, followed by the addition of 25 mL of TPP solution. The resulting mixture was then sonicated for 40 min at a temperature of 26 ± 1 °C and heated at 50 °C for 1 h. Finally, the magnetic Cs/MoS_2_/Fe_3_O_4_ nanocomposite was separated using a magnet, washed twice with ethanol and deionized water, and dried at 80 °C^30,33–36^.

### Characterization

To analyze the structure of the magnetics Cs/MoS_2_/Fe_3_O_4_ nanocomposite, various instruments were employed. The functional groups of the nanocomposite were examined using a Bruker Vertor-22 FTIR instrument from Germany. The dispersibility and size of the prepared nanocomposite were observed through Transmission Electron Microscopy (EM Philips EM 208S). The XRD pattern of the sorbent was obtained using a Rigaku-12 KW X-ray diffractometer. The magnetic properties of the sorbent were investigated using a vibrating sample magnetometer from Lake Shore Cryotronics in the USA. Additionally, Field Emission-Scanning Electron Microscopy (MIRA3 TESCAN, Czech Republic) was utilized to study the morphology of the sorbent. The analysis of PAHs was conducted using the Agilent GC/MS system (Model: 7890B, USA) following a previously established method^37^. Helium gas (99.99% purity) as the carrier gas with a flow rate of 1.8 mL/min was selected. The temperature program involved three steps. Firstly, it was set at 70 °C and then increased to 250 °C at a rate of 15 °C/min. Subsequently, it was further increased to 315 °C at a rate of 5 °C/min, and held at this temperature for 5 min. The MS was operated in full scan mode with an ion source temperature of 270 °C. It scanned a range of 50 to 320 m/z and utilized an ionization energy of 70 eV.

### Adsorption study

The magnetic Chitosan/MoS_2_ nanocomposite as a sorbent was used for removing PAHs in batch adsorption experiments. These experiments were performed using an Erlenmeyer flask (50 mL), and various operational parameters were investigated. The initial concentration of PAHs was 150 mg/L, and the dosage of the nanocomposite and the pH ranged from 0.01 to 0.06 g and 5.0 to 8.0 using NaOH or HCl aqueous solution with a concentration of 0.1 M, respectively. The contact time was fixed at range of 10 to 160 min, and the experiments were conducted at room temperature. To prepare the samples, a milk sample of 25.0 mL was spiked with a standard solution of PAHs, resulting in a final PAH concentration of 150 mg/L. Then, 0.04 g of the magnetic Chitosan/MoS_2_ nanocomposite was added to the spiked milk sample. The adsorption process took place at room temperature with continuous shaking at 150 rpm for 150 min. After completing the adsorption of PAHs, the sorbent was completely separated from the spiked milk sample solution using a magnet. The solution (1 µL) was injected into GC/MS for further analysis. The adsorption capacity (q_e_) and removal percentage (R%) of the PAH removal from the spiked milk sample solution were determined by the following equations:

1 $$R\%=\frac{{C}_{i}-{C}_{e}}{{C}_{i}}* 100$$

2 $${q}_{e}=\frac{\left({C}_{i}-{C}_{e}\right)* V}{M}$$

Here, C_e_ and C_i_ represent the final and initial concentrations of PAHs (mg/L), respectively, while V denotes the milk sample volume (mL), and M indicates the sorbent mass (g)^20,22,38,39^.

### Adsorption isotherms

In the experiments on adsorption isotherms, a quantity of sorbent (0.04 g) alongside a 25.0 mL of spiked milk sample containing PAHs at concentrations ranging from 20 to 150 mg/L. The mixture was shaken at 200 rpm for 3h. The adsorption data underwent adjustments through the utilization of Langmuir, Freundlich, Sips, and Dubinin- Adushkevich models^40^.

### Adsorption kinetics

The kinetic study was carried out using a spiked PAH solution (25.0 mL) with a concentration of 100 mg/L, and 0.04 g of sorbent. The experiments were conducted in a shaker operating at 200 rpm, maintaining a temperature of 25 ± 1 °C. Later, 20 µL of milk sample was extracted from the supernatant for each analysis over a total duration of 150 min. The rate constants for the adsorption of PAHs were determined by fitting the experimental data to the pseudo- second order and pseudo- first order models^40^.

### Ethics statement

The authors confirm they did not disturb dairy cows in any way, and raw milk sampling was purchased from markets (Neyshabur, Iran). We declare that all methods were carried out in accordance with relevant guidelines and regulations. The method of this study has been approved by the Ethics Committee of Neyshabur University of Medical Sciences (IR.NUMS.REC.1401.001).

## Results and discussion

### FTIR analysis

**Figure 1:**
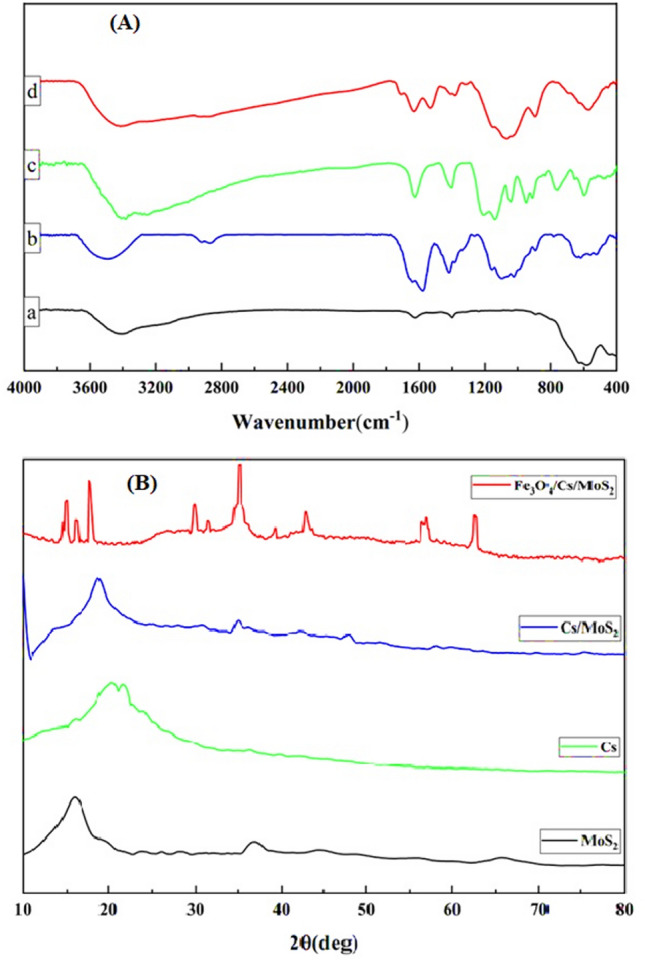
Fourier transform infrared spectra (**A**) of Fe_3_O_4_ nanoparticles (a), chitosan (b), (MoS_2_) (c) and Cs/MoS_2_/Fe_3_O_4_ nanocomposite (d) and XRD patterns (**B**) of CS, MoS_2_, CS/MoS_2_, and Cs/MoS_2_/Fe_3_O_4_ nanocomposite.

FTIR spectra were utilized to assess the chemical structures and functional groups of the sorbent. The FTIR spectrum of Fe_3_O_4_ (Fig. 1Aa) displayed peaks at 3439 cm^−1^ and 584 cm^−1^, which corresponded to the stretching vibration of free hydroxyl groups and Fe–O bonds, respectively^41,42^. On the other hand, the CS spectrum (Fig. 1Ab) showed a broad peak ranging from 3200–3550 cm^−1^, which was attributed to the stretching vibration of hydroxyl groups^43^. Additionally, the presence of −CH_2_− vibration is shown at 2872 and 2941 cm^−1^, while the peak at 1653 cm^−1^ indicated the presence of the stretching vibration of C–O groups^43–45^. The FTIR spectrum of the produced MoS_2_ is presented in Fig. 1Ac, indicating specific peaks at 639, 893, 1402, and 1622 cm^−1^. Furthermore, the peaks at 931 and 3182 cm^−1^ were related to the S–S bond and the broad stretching band of the O–H group, respectively^46^. It is worth noting that the characteristic stretching band of C=O presented in the amide group shifted towards a lower wavenumber, potentially because of the electrostatic interaction between the negative charge on the surface of MoS_2_ and positive charge of CS^46^. Lastly, in the FTIR spectrum of the Cs/MoS_2_/Fe_3_O_4_ nanocomposite pattern (Fig. 1Ad), the peak at 1599 cm^-1^ was attributed to the deformation vibration of the amine groups in CS. Additionally, the wide peak at 3423 cm^-1^ was formed by the stretching vibration of hydroxyls in Fe_3_O_4_ and CS^46,47^. These observed peaks collectively indicate the successful combination of Fe_3_O_4_, CS, and MoS_2_.

### XRD analysis

The XRD pattern was used to examine the crystalline structure of the produced adsorbent. In the case of CS, several peaks were observed at 2θ = 15.6°, 17.4°, and 21.8°, which are indicative of a semi-crystalline polymer. The peak at 2θ = 19.97° corresponds to the crystalline chitosan framework, confirming the presence of biopolymer backbone chains^47^. These XRD results are consistent with previous findings on CS diffraction patterns^48^. Moving on to the XRD pattern of MoS_2_, multiple peaks were identified at 2θ angles of 14°, 28°, 32°, 33°, 35°, 39°, 44°, 49°, 56°, 60°, and 70°, corresponding to lattice plane (hkl) values of (002), (004), (100), (101), (102), (103), (006), (105), (106), (008), and (108), respectively. These diffraction peaks confirm the hexagonal structure of MoS_2_, matching well with the JCPDS card No. 37–1492^49^. In the CS/MoS_2_ composite, the XRD pattern (Fig. 1B) shows a slight shift in the peak position of the high-intensity (002) lattice plane of MoS_2_ towards higher angles compared to pristine MoS_2_. This shift is attributed to the modification of the MoS_2_ crystal lattice by functionalized CS, providing further evidence for the formation of the CS/MoS_2_ nanocomposite. However, in the XRD pattern of the Cs/MoS_2_/Fe_3_O_4_ nanocomposite, the diffraction peaks of MoS_2_ are not clearly discernible due to their masking by the strong peaks of Fe_3_O_4_. This observation aligns with a previous study^50^.

### TEM and FE-SEM studies

**Figure 2:**
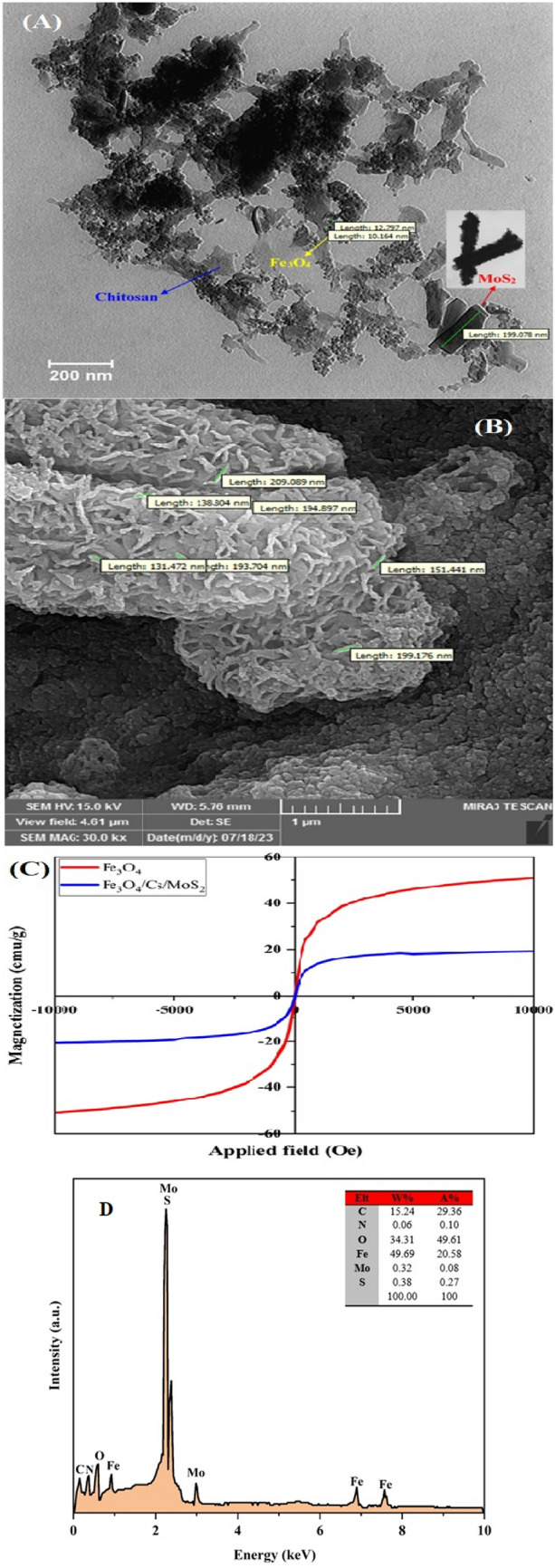
TEM (**A**) and SEM (**B**) images of Cs/MoS_2_/Fe_3_O_4_ nanocomposite, and VSM patterns (**C**) of Fe_3_O_4_ nanoparticle and Cs/MoS_2_/Fe_3_O_4_ nanocomposite, and EDX pattern (**D**) of the CS/MoS_2_/Fe_3_O_4_ nanocomposite.

**Figure 3:**
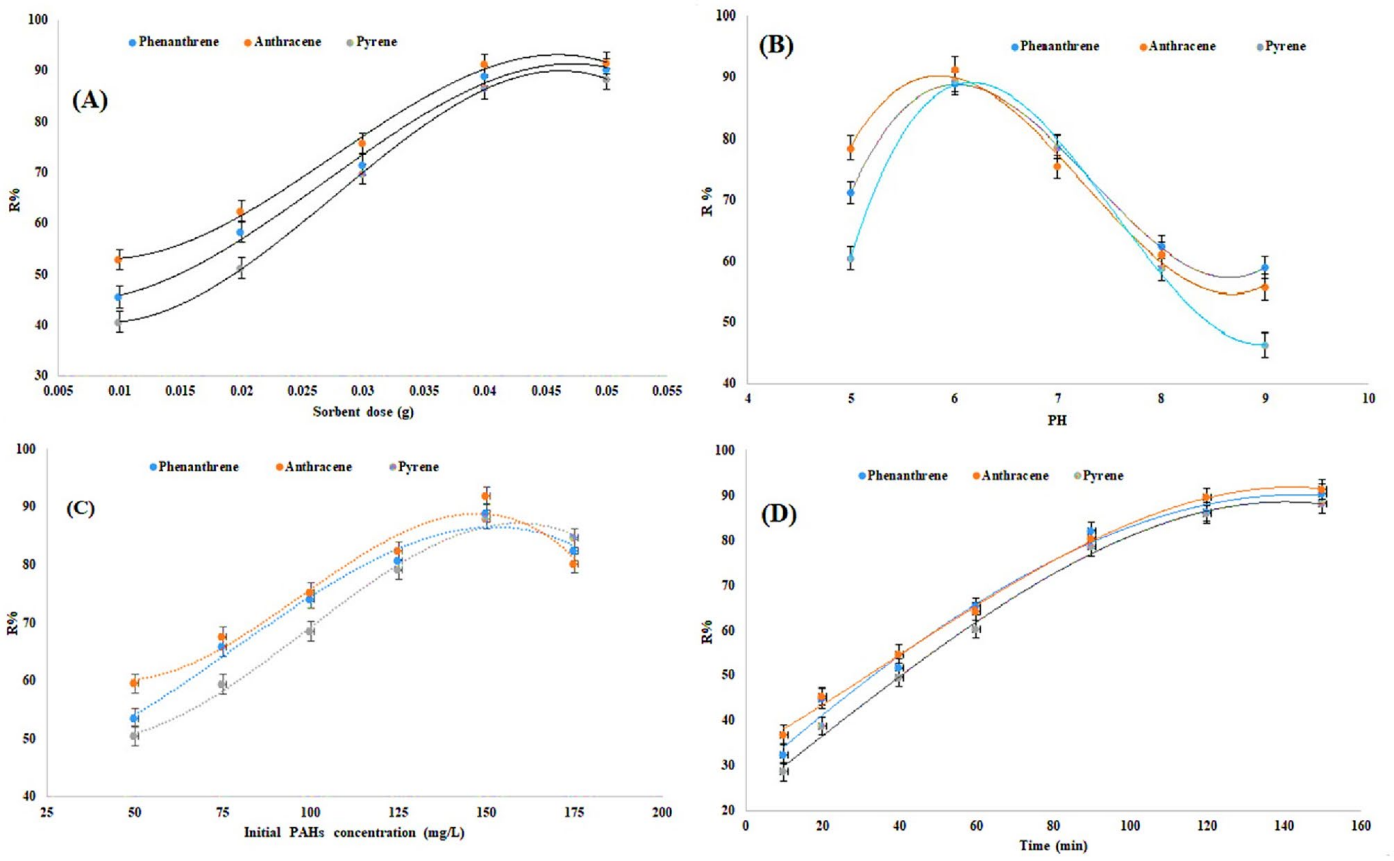
Effects of Cs/MoS_2_/Fe_3_O_4_ nanocomposite dose (**A**), pH of the solution (**B**), initial PAHs concentration (**C**), and contact time (**D**) on percentage removal of phenanthrene, anthracene, and pyrene from milk.

**Figure 4:**
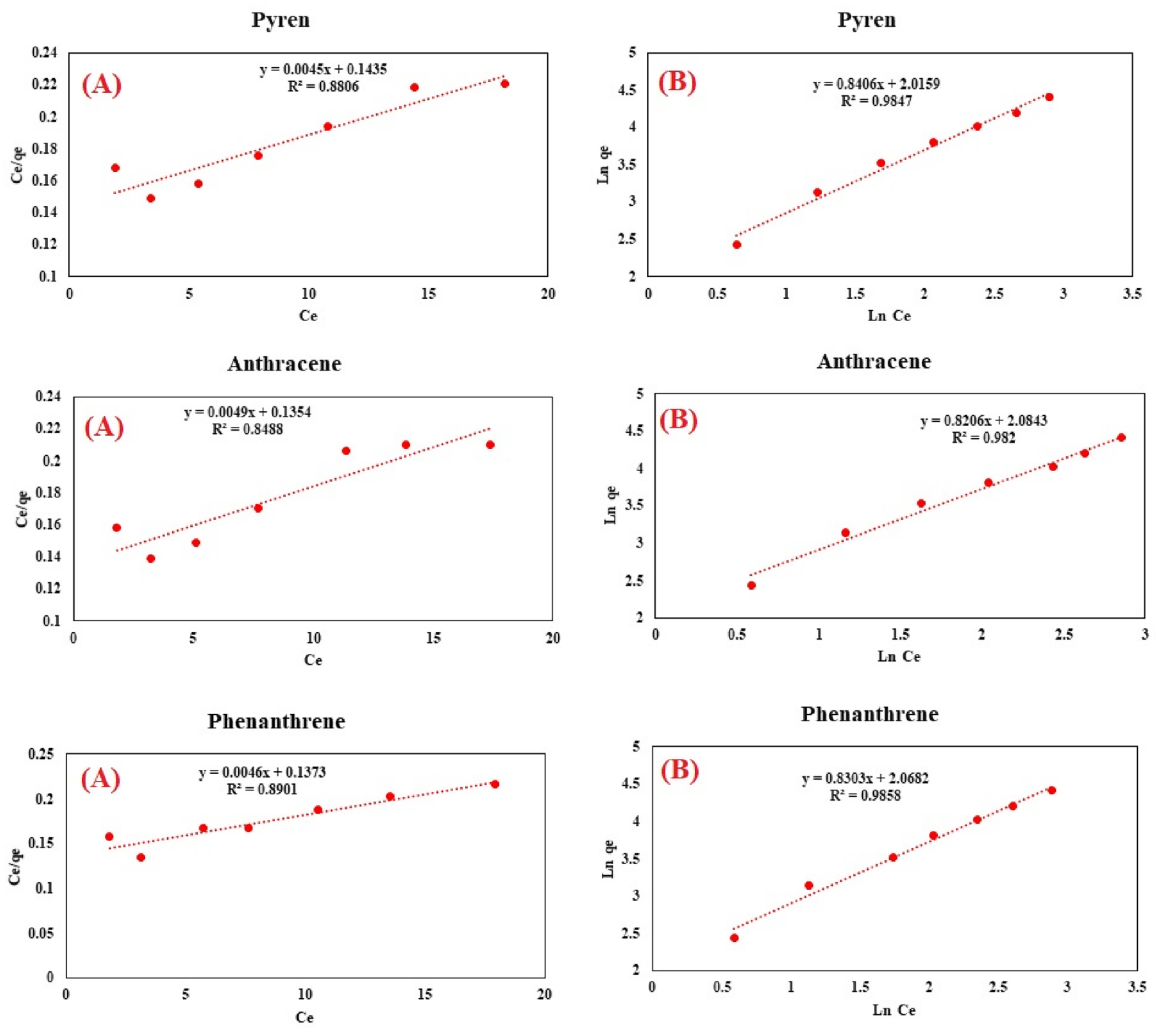
Langmuir (**A**) and Freundlich (**B**) sorption diagrams for pyrene, anthracene, and phenanthrene.

The nanocomposite's morphology and size were analyzed using TEM. In Fig. 2A, a typical TEM image illustrates the well-dispersed Fe_3_O_4_ particles with an average diameter of approximately 10–12 nm, as clearly shown in the inset of Fig. 3. Upon combining with MoS_2_, a distinct uniform core–shell structure is observed (Fig. 2A), where crumpled few-layer MoS_2_ nanosheets form a thin shell around the Fe_3_O_4_, with a length of about 199 nm. The layered structure of MoS_2_ is evident, with individual layers discernible in bright and darker sections from the layer cross-section^51^. At higher magnification, the TEM reveals that some Fe_3_O_4_ and CS are bound to the surface of the MoS_2_ nanosheets. Additionally, the nanocomposite's morphology and surface uniformity were examined using FE-SEM images. As depicted in Fig. 4, the FE-SEM images show the flake-like MoS_2_ structure deposited on Fe_3_O_4_ nanoparticles in the synthesized nanocomposite. Furthermore, Fig. 2b displays the spherical and regular Fe_3_O_4_ nanoparticles effectively loaded onto CS/MoS_2_, indicating the formation of the CS/MoS_2_/Fe_3_O_4_ nanocomposite.

The energy-dispersive X-ray spectroscopy (EDX) pattern of the CS/MoS_2_/Fe_3_O_4_ nanocomposite is depicted in Fig. 2d. This pattern reveals that the composite material consists of carbon, oxygen, iron, sulfur, nitrogen and molybdian elements, with weight percentages of 15.24%, 34.31%, 49.69%, 0.38%, 0.06% and 0.32%, respectively. Notably, carbon and molybdian exhibit the highest and lowest weight percentages within the sorbent structure.

### VSM analysis

The magnetic properties of the CS/MoS_2_/Fe_3_O = nanocomposite were analyzed using hysteresis curves obtained through VSM, which revealed zero coercivity and remanence, indicating the nanocomposite has the super-magnetism property. As illustrated in Fig. 2C, Fe_3_O_4_ displayed a high degree of magnetization (~ 50 emu/g). Besides, the magnetization value considerably decreased to around 18 emu/g upon coating Fe_3_O_4_ with CS/MoS_2_ (Fig. 2C). This phenomenon can be attributed to the presence of additional materials in the nanocomposite, which can alter the magnetic properties of Fe_3_O_4_^35^. It is worth noting that magnetic nanocomposites have potential applications as adsorbents for removing toxic agents from aqueous solutions due to their high surface-to-volume ratio, water repellency, fast diffusion, and super-magnetism^51,52^.

### Adsorption study

#### Effect of Cs/MoS_2_/Fe_3_O_4_ nanocomposite dose

Determining the optimal dosage of Cs/MoS_2_/Fe_3_O_4_ nanocomposite for achieving maximum adsorption of PAHs is a crucial factor in adsorption studies^53,54^. In this study, the impact of varying dosages of Cs/MoS_2_/Fe_3_O_4_ nanocomposite ranging from 0.01 to 0.05 g on the adsorption of PAHs (phenanthrene, anthracene, and pyrene) was extensively investigated and analyzed (refer to Fig. 3A). The results revealed a significant trend, where the adsorption efficiencies of the PAHs increased with increasing dosage of the nanocomposite from 0.01 to 0.045 g, eventually reaching a plateau. This behavior can be attributed to the availability of active sites on the surface of the sorbent that play a crucial role in the interaction with the PAHs. Thus, an increase in the sorbent dosage led to a corresponding increase in the removal of PAHs due to an enhanced number of active sites available for the adsorption process. These findings are consistent with the results reported by Zhou et al. in their investigation of the impact of nanocomposite dosage on the maximum adsorption of PAHs^55^. Therefore, based on these collective findings, a sorbent dosage of 0.045 g was selected for further detailed investigation and analysis in subsequent experiments.

#### Effect of solution pH

The pH is an important parameter to consider when studying liquid-phase adsorption^55^. Figure 3B depicts the adsorption of phenanthrene, anthracene, and pyrene from milk samples using Cs/MoS_2_/Fe_3_O_4_ nanocomposite at various pH values ranging from 5 to 8. The adsorption percentage of these PAHs increased within the pH range of 3 to 6 but decreased afterward. This can be attributed to the favorable interaction between the aromatic rings of the PAHs and the ion-pair of amines or hydroxyl groups in chitosan, which is present in the sorbent and aids in PAH removal. At low pH levels, chitosan in the sorbent dissolved in the solution, resulting in the protonation of its amine groups. As a consequence, the negative charge on the sorbent surface decreased, limiting its interaction with the delocalized π electrons in the aromatic rings of PAHs. Conversely, at high pH levels, hydroxide ions in the solution competed with the sorbent for interaction with PAHs. This competition reduced the adsorption of PAHs on the sorbent's surface.

#### Effect of initial PAH concentration

The efficiency of PAH removal was evaluated using the synthesized nanocomposite at different concentrations (ranging from 50 to 175 mg/L), and the results are depicted in Fig. 3C. The highest removal efficiency was observed when the PAH concentration was at 150 mg/L. This finding can be attributed to the relationship between the PAH concentration and the number of active sites available on the surface of the nanocomposite^56^. When the number of active sites on the sorbent surface becomes saturated with PAHs, they are no longer adsorbed. Thus, as long as there are active sites available, there is potential for interaction with PAHs. Consequently, the removal of PAHs increased as the PAH concentration increased up to 150 mg/L and then decreased thereafter. Therefore, a PAH concentration of 150 mg/L was chosen as the optimal value for further investigation.

#### Effect of contact time

The duration of contact between PAHs and the sorbent is a crucial factor that influences the efficiency of adsorption^57^. Contact time refers to the minimum duration required to achieve a consistent concentration, known as the adsorption equilibrium stage. Once this equilibrium is reached, the concentration of PAHs in the solution remains stable because the amount of PAHs absorbed on the sorbent's surface equals the amount desorbed into the sample solution. In this study, the influence of contact time on the adsorption efficiency of PAHs was examined at room temperature over various durations (ranging from 10 to 160 min). As shown in Fig. 3D, the adsorption of PAHs increased up to 150 min and then remained constant with further increases in time. This suggests that PAHs reached equilibrium on the sorbent's surface after 150 min. Therefore, a contact time of 150 min was chosen as the optimal value for further investigation.

### Adsorption isotherms and kinetic study

In order to assess the efficiency of the sorbent's adsorption, we examined the equilibrium adsorption values of phenanthrene, anthracene, and pyrene within the concentration range of 20 to 150 mg/L on the sorbent (0.045 g) using the Langmuir and Freundlich models. The Langmuir (Eq. 3), Freundlich (Eq. 4), Sips (Eq. 5), and Dubinin-Adushkevich (Eqs. 6 and 7) isotherm models can be represented by the following equations^21,58^:

3 $$\frac{{\text{C}}_{\text{e}}}{{\text{q}}_{\text{e}}}=\frac{{\text{C}}_{\text{e}}}{{\text{q}}_{\text{max}}}+\frac{1}{{\text{bq}}_{\text{max}}}$$

4 $${\text{ln q}}_{\text{e}}={\text{ln k}}_{\text{f}}+\frac{1}{\text{n}}{\text{ln C}}_{\text{e}}$$

5 $$\text{ln}\left(\frac{{q}_{mS}}{{q}_{e}}\right)=-\beta \,\text{ln}\left({C}_{e}\right)+\text{ln} {K}_{s}$$

6 $$ln{q}_{e}=ln{q}_{mDR}-{K}_{DR}{\varepsilon }^{2}$$

7 $$\varepsilon =RTln(1+\frac{1}{{C}_{e}})$$

Here, C_e_ and q_e_ represent the initial concentration of phenanthrene, anthracene, and pyrene (mg/L) spiked in milk samples, and the equilibrium absorption amount of the sorbent (mg/g), respectively. Besides, n, k_f_, b, and q_max_ denote the adsorption intensity, Freundlich constants (mg/g), Langmuir constant (L/mg), and maximum monolayer adsorption capacity (mg/g), respectively. Besides, K_s_, q_MS_ and β present equilibrium constant (L/mg), maximum adsorption capacity(mg/g), and model exponent of the Sips. K_DR_ (mol^2^/kJ), q_mDR_ (mg/g), and ε denote the adsorption energy constant, capacity of saturation theory, and potential Polanyi, respectively. Also, ε calculates using Eq. (8). The regression coefficient (R^2^) obtained for each graph was utilized to select the appropriate isotherm model. Based on the results in Table 1, the R^2^ values obtained from the Langmuir and Dubinin- Adushkevich equations were lower than those from the Freundlich and Sips equations for the adsorption of PAHs, showing that the Freundlich and Sips models effectively describes the absorption of these compounds. These findings align with the results presented in the TEM and FE-SEM images of Cs/MoS_2_/Fe_3_O_4_ nanocomposite as the sorbent (Fig. 2A, B), which demonstrate heterogeneity on the sorbent surface. The desirability of the adsorption process was evaluated using the adsorption intensity (n). Suitable absorption of PAHs on the sorbent occurs when the n-value falls between 1 and 10. The n-values of 1.49, 1.20, and 1.20 from the Freundlich equation for the adsorption of pyrene, anthracene, and phenanthrene, respectively, confirm that their adsorption on the produced nanocomposites is satisfactory. Additionally, the K_F_ values exceeding 8.03 obtained for the adsorption of PAHs indicate a high affinity for their adsorption process on the sorbent surface. Besides, a high value of β (β > 1) for the adsorption of PAHs in the sorbent obtained from Sips model indicated that interactions between PAHs and the sorbent were took place. Also, these interactions are strong because K_s_ are high. The maximum adsorption capacity (q_mS_) of the sorbent is in the range of 83.456 -83.709 mg/g for removing PAHs. The Langmuir equation slope was utilized to calculate the maximum adsorption capacity (q_max_) of Cs/MoS_2_/Fe_3_O_4_ nanocomposite to adsorb PAHs. The maximum absorption capacity to adsorb phenanthrene, anthracene, and pyrene on the produced nanocomposites was 217, 204, and 222 mg/g, respectively.

**Table 1:** Adsorption isotherms for removing PAHs by Cs/MoS_2_/Fe_3_O_4_ nanocomposite.

Isotherm model	PAH	Equation	Parameters		R^2^
Langmuir model	Pyrene	Y = 0.0045x + 0.1435	q_max_ (mg g^−1^)	222.2	0.881
			b (L mg^−1^)	0.031	
	Anthracene	Y = 0.0049x + 0.1354	q_max_ (mg g^−1^)	204.08	0.849
			b (L mg^−1^)	0.036	
	Phenanthrene	Y = 0.0046x + 0.1373	q_max_ (mg g^−1^)	217.39	0.890
			b (L mg^−1^)	0.033	
Freundlich model	Pyrene	Y = 0.8406x + 2.0159	K_f_ (mg g^−1^)	10.85	0.986
			n	1.49	
	Anthracene	Y = 0.8206x + 2.0843	K_f_ (mg g^−1^)	8.03	0.982
			N	1.20	
	Phenanthrene	Y = 0.8303x + 2.0682	K_f_ (mg g^−1^)	7.91	0.986
			N	1.20	
Dubinin Radushkevich	Pyrene	Y = −0.2495x + 5.8612	K_DR_	0.2495	0.9429
			q_mDR_ (mg g^−1^)	351.145	
	Anthracene	Y = −0.231x + 5.7349	K_DR_	0.231	0.9413
			q_mDR_ (mg g^−1^)	309.482	
	Phenanthrene	Y = −0.2297x + 5.7236	K_DR_	0.2297	0.9354
			q_mDR_ (mg g^−1^)	306.00	
Sips	Pyrene	Y = −1.1715x + 2.851	K_S_ (mL mg^−1^)	17.305	0.9847
			Β	1.1715	
			q_mS_(mg g^−1^)	83.456	
	Anthracene	Y = −1.1968x + 2.8384	K_S_ (mL mg^−1^)	17.09	0.982
			Β	1.1968	
			q_mS_ (mg g^−1^)	83.709	
	Phenanthrene	Y = −1.1873x + 2.8267	K_S_ (mL mg^−1^)	16.890	0.9858
			Β	1.1873	
			q_mS_ (mg g^−1^)	83.614	

The utilization of the newly created nanocomposite as a sorbent for eliminating PAHs comes with several benefits, one of which is its elevated surface-to-volume ratio. As a result, only a small quantity of sorbent is necessary, and optimal conditions lead to high adsorption capacity and suitable interaction with PAHs ^59^. To analyze the kinetics of the adsorption process and fit the adsorption data over time, different kinetic models have been employed, such as the pseudo-first-order (Eq. 8), pseudo-second-order (Eq. 9) and intra-particle diffusion kinetic (Eq. 10) models. The linear forms of these model equations used in the kinetic study are outlined below^22,60,61^:

8 $$\text{log}\left({q}_{e}-{q}_{t}\right)= \text{log} {q}_{e}- \frac{{k}_{1}t}{2.303}$$

9 $$\frac{\text{t}}{{\text{q}}_{\text{t}}}=\frac{1}{{\text{k}}_{2}{\text{q}}_{\text{e}}^{2}}+\frac{1}{{\text{q}}_{\text{e}}}\text{t}$$

10 $${\text{q}}_{\text{t}}={\text{k}}_{\text{i}} * {\text{t}}^\frac{1}{2}+C$$

**Figure 5:**
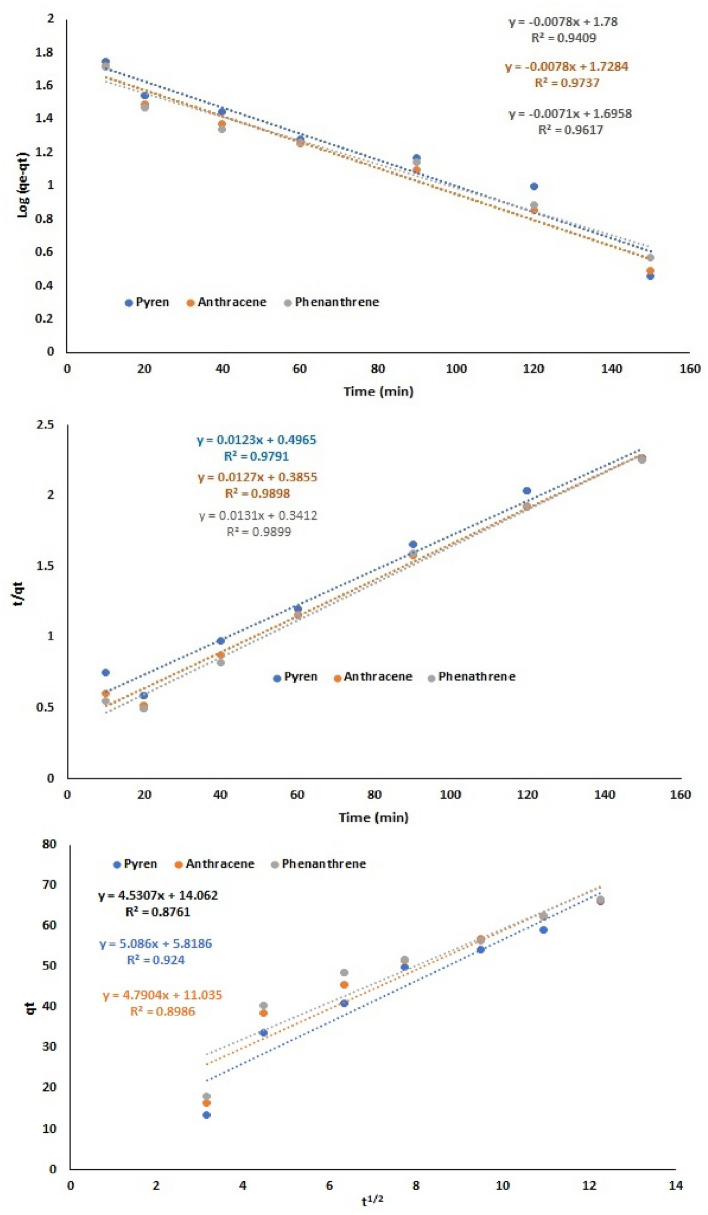
The effect of contact time on PAH removal by the Cs/MoS_2_/Fe_3_O_4_ nanocomposite according to the pseudo-first-order kinetic model (**A**), pseudo-second-order kinetic model (**B**), and intra-particle diffusion kinetic model (**C**).

These equations involve the variables q_e_ (mg/g), q_t_ (mg/g), and k, representing the amount of adsorbed PAHs at equilibrium time and at time t (min), and the rate constant, respectively. The impact of adsorption time was examined to assess and determine the appropriate kinetic models for the elimination of PAHs using the newly developed nanocomposites. The results for PAH removal are illustrated in Fig. 5. Generally, the adsorption of PAHs adheres to a pseudo-second-order kinetic model, as indicated by higher correlation coefficient values compared to other kinetic models. The q_e_ values and rate constants for adsorbing PAH were obtained from the equation and presented in Table 2. Moreover, the findings demonstrate that the removal percentage (R%) increases until approximately 150 min of adsorption time. This suggests that the removal process is rapid with an equilibrium time under 150 min for PAHs. The outcomes can be attributed to the effective removal process facilitated by the interaction between functional groups on the sorbent surface and those in PAHs^21^. Additionally, the diffusion of metal ions into the sorbent pores is significantly reduced. The low correlation coefficient values for the intra-particle diffusion model further confirm that PAH adsorption occurs through physical or chemical interactions between the sorbent and PAHs. Notably, the penetration of PAHs into the sorbent pores is minimal.

**Table 2:** Adsorption kinetics for removing PAHs by Cs/MoS_2_/Fe_3_O_4_ nanocomposite.

Kinetic model	PAH	Equation	Parameters		R^2^
Pseudo- first- order model	Pyrene	Y = −0.0078x + 1.78	q_e_ (mg g^−1^)	60.25	0.941
			K_1_ (min^−1^)	0.018	
	Anthracene	Y = −0.0078x + 1.7284	q_e_ (mg g^−1^)	53.50	0.974
			K_1_ (min^−1^)	0.017	
	Phenanthrene	Y = −0.0071x + 1.6958	q_e_ (mg g^−1^)	49.63	0.962
			K_1_ (min^−1^)	0.016	
Pseudo- second-order model	Pyrene	Y = 0.0123x + 0.4965	q_e_ (mg g^−1^)	81.30	0.979
			K_2_ × 10^−4^ (g mg^−1^ min^−1^)	3.05	
	Anthracene	Y = 0.0127x + 0.3855	q_e_ (mg g^−1^)	78.74	0.990
			K_2_ × 10^−4^ (g mg^−1^ min^−1^)	4.18	
	Phenanthrene	Y = 0.0131x + 0.3412	q_e_ (mg g^−1^)	76.33	0.990
			K_2_ × 10^−4^ (g mg^−1^ min^−1^)	5.03	
Intra-particle diffusion model	Pyrene	Y = 5.086x + 5.8186	C (mg g^−1^)	5.8186	0.924
			K_i_ (mg g^−1^ min^−1/2^)	5.086	
	Anthracene	Y = 4.7904x + 11.035	C (mg g^−1^)	11.035	0.8986
			K_i_ (mg g^−1^ min^−1/2^)	4.7904	
	Phenanthrene	Y = 4.5307x + 14.062	C (mg g^−1^)	14.062	0.8761
			K_i_ (mg g^−1^ min^−1/2^)	4.5307	

### The sorbent reusability

The reusability of sorbents holds significant importance for several reasons. Firstly, it mitigates costs associated with disposal and replacement, rendering the remediation process economically viable, particularly for large-scale endeavors. Secondly, it curtails waste production, thereby promoting environmental preservation and sustainability. Thirdly, it amplifies the efficiency of PAH removal by optimizing the utilization of sorbent material across multiple cycles, thereby enhancing overall remediation efficacy. Consequently, the reusability of magnetic chitosan/MoS_2_ nanocomposite sorbents for PAH removal was investigated. To achieve this, the sorbent underwent washing with methanol in triplicate to desorb PAHs from the sorbent surface. Subsequently, the sorbent was dried at 50°C for reuse. The results, as presented in Table 3, indicate that the sorbent can effectively remove PAHs through four adsorption–desorption cycles with minimal variance in R%. Experimental findings suggest that the sorbent maintains high adsorption capacity and magnetic retrievability even after multiple cycles, underscoring its potential for repeated use in PAH remediation applications.

**Table 3:** Comparison of the various sorbents for removing PAHs.

Sorbent	Adsorption capacity (mg/g)	Ref
Graphene nanosheets	150.2	^71^
Graphene nanosheets	116 (PHEN), 123 (PYR)	^67^
Exfoliated grapheme	24.1 (PHEN)	^68^
Orange rind activated carbon	70.92	^69^
Modified palm shell activated carbon by KOH	96.54	^72^
Fe_3_O_4_-SiO_2_-2DMDPS	47.315	^70^
Fe_3_O_4_-SiO_2_	34.67	^70^
Rice husk activated carbon	50.4	^73^
In organo-organo-bentonite	13.32	^74^
Cs/MoS_2_/Fe_3_O_4_ nanocomposite	222 (pyrene), 204 (anthracene), 217 (phenanthrene)	This work

### Comparison with other sorbents

As mentioned in the preceding sections, PAHs are significant environmental pollutants with adverse effects on human health^62^. Their presence has been documented in various food products, including milk, raising concerns due to its widespread consumption^12,63^. Various methods are available for eliminating PAHs from solid and liquid foods^64^. Among these approaches, the adsorption method shows promise as a cost-effective and environmentally friendly option^65^. In recent years, there has been growing interest in magnetic nanocomposite-based sorbents due to their superparamagnetic properties, which enable magnetic field-assisted separation and enrichment^66^. The adsorption efficiency of the developed nanocomposites was compared to that of other magnetic nanocomposite sorbents, such as graphene nanosheets^67^, exfoliated graphene^68^, orange rind activated carbon^69^, Fe_3_O_4_-SiO_2_-2DMDPS, and Fe_3_O_4_-SiO_2_^70^ (Table 3). The findings indicate that the sorbent demonstrates the highest adsorption capacity among the prepared sorbents for PAH removal.

## Conclusion

After synthesizing and characterizing a magnetic nanocomposite comprised of Cs/MoS_2_/Fe_3_O_4_, we successfully utilized it as an efficient sorbent for eliminating PAHs from milk. Through comprehensive characterization techniques including XRD, FTIR, FE-SEM, TEM, and VSM, we confirmed the successful coating of Cs onto the MoS_2_/Fe_3_O_4_ nanocomposite. Various factors affecting the PAH adsorption process, such as nanocomposite dosage, initial PAH concentration, pH, and contact time were systematically investigated. Our findings revealed that the lowest and highest removal efficiencies were observed at nanocomposite doses of 0.01 g and 0.045 g, respectively. The optimal conditions for maximum PAH removal were identified as a pH of 6.0, a sorbent amount of 0.045 g, an initial PAH concentration of 150 mg/L, and a contact time of 150 min. Furthermore, the adsorption data for PAH removal using the prepared composite exhibited excellent agreement with the Freundlich isotherm model and the pseudo-second-order kinetics model, indicating a superior fit compared to the Langmuir isotherm model and the pseudo-first-order kinetics model. The procedure for removing PAHs utilizing the sorbent exhibits several notable strengths. Firstly, employing a magnetic chitosan/MoS_2_ nanocomposite absorbent for PAH removal introduces a pioneering method, which holds promise for superior efficacy compared to conventional techniques. Additionally, integrating MoS_2_ nanoparticles into the chitosan matrix has the potential to enhance adsorption properties due to the abundant surface area and functional groups inherent in MoS_2_. Moreover, the inclusion of magnetic nanoparticles enables effortless separation of the sorbent from the solution using an external magnetic field, streamlining the recovery process and reducing operational expenses. Furthermore, chitosan's biodegradability and environmental compatibility bolster the sustainability of the sorbent material. However, the procedure does have certain limitations. Chief among them is the scalability of the synthesis process for the magnetic chitosan/MoS_2_ nanocomposite, which may pose challenges, particularly for large-scale applications, potentially impeding its industrial feasibility. Furthermore, while chitosan is regarded as environmentally benign, concerns arise regarding the potential release of MoS2 nanoparticles during the absorption process, necessitating comprehensive risk assessment studies to evaluate their long-term environmental impact and toxicity.

## Data Availability

The datasets used during the current study available from the corresponding author on reasonable request.

## References

[1] Lawal AT (2017). Polycyclic aromatic hydrocarbons. A review. Cogent. Environ. Sci..

[2] Liaud C, Millet M, Le Calvé S (2015). An analytical method coupling accelerated solvent extraction and HPLC-fluorescence for the quantification of particle-bound PAHs in indoor air sampled with a 3-stages cascade impactor. Talanta.

[3] Wang M, Cheng C, Liu C, Yang Y (2018). Hollow fiber supported ionic liquids liquid-phase micro-extraction followed by high-performance liquid chromatography for the determination of polycyclic aromatic hydrocarbons in milk samples. J. Chromatogr. Sci..

[4] Inbaraj BS, Sridhar K, Chen B-H (2021). Removal of polycyclic aromatic hydrocarbons from water by magnetic activated carbon nanocomposite from green tea waste. J. Hazard. Mater..

[5] Schneider, I. L.* et al.* FTIR analysis and evaluation of carcinogenic and mutagenic risks of nitro-polycyclic aromatic hydrocarbons in PM1.0. *Sci. Total Environ.* **541**, 1151–1160 (2016). 10.1016/j.scitotenv.2015.09.142 26473715

[6] IARC. *International Agency for Research on Cancer*. Vol. 1–121. http://monographs.iarc.fr/ENG/Classification. Accessed 17 Feb 2021 (2021).

[7] Organization WH. *International Agency for Research on Cancer*. Accessed 10 Oct 2021 (2021).

[8] Duan X (2016). Dietary intake polycyclic aromatic hydrocarbons (PAHs) and associated cancer risk in a cohort of Chinese urban adults: Inter-and intra-individual variability. Chemosphere.

[9] Singh L, Agarwal T (2018). Polycyclic aromatic hydrocarbons in diet: Concern for public health. Trends Food Sci. Technol..

[10] Martorell I (2010). Polycyclic aromatic hydrocarbons (PAH) in foods and estimated PAH intake by the population of Catalonia, Spain: Temporal trend. Environ. Int..

[11] Iwegbue CM, Onyonyewoma UA, Bassey FI, Nwajei GE, Martincigh BS (2015). Concentrations and health risk of polycyclic aromatic hydrocarbons in some brands of biscuits in the Nigerian market. Hum. Ecol. Risk Assess. Int. J..

[12] Shariatifar N (2020). Levels of polycyclic aromatic hydrocarbons in milk and milk powder samples and their likely risk assessment in Iranian population. J. Food Compos. Anal..

[13] Shojaeimeher, S., Babashahi, M., Shokri, S., Mirlohi, M. & Zeinali, T. Optimizing the production of probiotic yogurt as a new functional food for diabetics with favorable sensory properties using the response surface methodology. *Probiot. Antimicrob. Proteins* 1–13 (2023). 10.1007/s12602-023-10051-z 36928935

[14] Akbari-Adergani B (2021). GC–MS determination of the content of polycyclic aromatic hydrocarbons in bread and potato Tahdig prepared with the common edible oil. Environ. Monit. Assess.

[15] Shavali-Gilani P, Yazdanfar N, Jahed-Khaniki G, Molaee-Aghaee E, Sadighara P (2023). The effect of flavorings on PAHs level in the roasted sunflower seeds. Sci. Rep..

[16] Kim D-Y, Kim B, Shin H-S (2021). Reduction of polycyclic aromatic hydrocarbons (PAHs) in sesame oil using cellulosic aerogel. Foods.

[17] Wang Z, Ng K, Warner RD, Stockmann R, Fang Z (2022). Reduction strategies for polycyclic aromatic hydrocarbons in processed foods. Comprehens. Rev. Food Sci. Food Saf..

[18] Yousefi M (2022). Effect of time and incubation temperature on ability of probiotics for removal of polycyclic aromatic hydrocarbon in phosphate buffer saline. J. Microbiol. Biotechnol. Food Sci..

[19] Ghorbani, M., Seyedin, O. & Aghamohammadhassan, M. Adsorptive removal of lead (II) ion from water and wastewater media using carbon-based nanomaterials as unique sorbents: A review. *J. Environ. Manag.* **254**, 109814 10.1016/j.jenvman.2019.109814 (2020). 10.1016/j.jenvman.2019.109814 31726282

[20] Gordi, Z., Ghorbani, M. & Ahmadian Khakhiyani, M. Adsorptive removal of enrofloxacin with magnetic functionalized graphene oxide @ metal‐organic frameworks employing D‐optimal mixture design. *Water Environ. Res.* **92**, 1935–1947 10.1002/wer.1346 (2020). 10.1002/wer.1346 32319707

[21] Ghorbani, M., Shams, A., Seyedin, O. & Afshar Lahoori, N. Magnetic ethylene diamine-functionalized graphene oxide as novel sorbent for removal of lead and cadmium ions from wastewater samples. *Environ. Sci. Pollut. Res.* **25**, 5655–5667 10.1007/s11356-017-0929-7 (2017). 10.1007/s11356-017-0929-7 29222663

[22] Ghorbani, M., Ariavand, S., Aghamohammadhasan, M. & Seyedin, O. Synthesis and optimization of a green and efficient sorbent for removal of three heavy metal ions from wastewater samples: Kinetic, thermodynamic, and isotherm studies. *J. Iran. Chem. Soc.* **18**, 1947–1963 10.1007/s13738-021-02161-8 (2021).

[23] Khosravi-Darani, K., Gomes da Cruz, A., Shamloo, E., Abdimoghaddam, Z. & Mozafari, M. Green synthesis of metallic nanoparticles using algae and microalgae. *Lett. Appl. Nanobiosci.* **8**, 666–670 (2019).

[24] Borji H, Ayoub GM, Al-Hindi M, Malaeb L, Hamdan HZ (2020). Nanotechnology to remove polychlorinated biphenyls and polycyclic aromatic hydrocarbons from water: A review. Environ. Chem. Lett..

[25] Ogunfowora LA, Iwuozor KO, Ighalo JO, Igwegbe CA (2021). Trends in the treatment of aquaculture effluents using nanotechnology. Clean. Mater..

[26] Bayramoglu G, Arica MY (2018). Adsorption of Congo Red dye by native amine and carboxyl modified biomass of *Funalia trogii*: Isotherms, kinetics and thermodynamics mechanisms. Korean J. Chem. Eng..

[27] Bayramoglu G, Kunduzcu G, Arica MY (2020). Preparation and characterization of strong cation exchange terpolymer resin as effective adsorbent for removal of disperse dyes. Polymer Eng. Sci..

[28] Khah MH, Jamshidi P, Shemirani F (2021). Applying Fe_3_O_4_-MoS_2_-chitosan nanocomposite to preconcentrate heavy metals from dairy products prior quantifying by FAAS. Res. Chem. Intermediates.

[29] Cao W, Yue L, Zhang Y, Wang Z (2022). Photodynamic chitosan functionalized MoS_2_ nanocomposite with enhanced and broad-spectrum antibacterial activity. Carbohydr. Polymers.

[30] Kasinathan K (2020). Synthesis of biogenic chitosan-functionalized 2D layered MoS2 hybrid nanocomposite and its performance in pharmaceutical applications: In-vitro antibacterial and anticancer activity. Int. J. Biol. Macromol..

[31] Zeb MA, Shah J, Jan MR (2022). Biopolymer magnetic chitosan–graphene oxide composite for removal and extraction of aromatic amines from aqueous samples. J. Chem. Technol. Biotechnol..

[32] Zhang D (2019). Highly effective shielding of electromagnetic waves in MoS_2_ nanosheets synthesized by a hydrothermal method. J. Phys. Chem. Solids.

[33] Rezagholizade-shirvan A, Najafi MF, Behmadi H, Masrournia M (2022). Preparation of nano-composites based on curcumin/chitosan-PVA-alginate to improve stability, antioxidant, antibacterial and anticancer activity of curcumin. Inorgan. Chem. Commun..

[34] Rezagholizade-shirvan, A., Masrournia, M., Fathi Najafi, M. & Behmadi, H. Synthesis and characterization of nanoparticles based on chitosan-biopolymers systems as nanocarrier agents for curcumin: study on pharmaceutical and environmental applications. *Polymer Bull.* **80**, 1495–1517 (2023).

[35] Shokri S (2023). Synthesis and characterization of a novel magnetic chitosan–nickel ferrite nanocomposite for antibacterial and antioxidant properties. Sci. Rep..

[36] Farhadi L (2022). Green synthesis of chitosan-coated silver nanoparticle, characterization, antimicrobial activities, and cytotoxicity analysis in cancerous and normal cell lines. J. Inorgan. Organomet. Polymers Mater..

[37] Kandil A, Halawa AA, Shata R, Mohamed SS, Al-Ashmawy MA (2023). Detection of polycyclic aromatic hydrocarbons concentrations in Egyptian raw and sterile milk. J. Adv. Vet. Res..

[38] Rose PK, Kumar R, Kumar R, Kumar M, Sharma P (2023). Congo red dye adsorption onto cationic amino-modified walnut shell: Characterization, RSM optimization, isotherms, kinetics, and mechanism studies. Groundw. Sustain. Dev..

[39] Shokri S (2024). Modeling sunset yellow removal from fruit juice samples by a novel chitosan-nickel ferrite nano sorbent. Sci. Rep..

[40] Solano RA, De León LD, De Ávila G, Herrera AP (2021). Polycyclic aromatic hydrocarbons (PAHs) adsorption from aqueous solution using chitosan beads modified with thiourea, TiO_2_ and Fe_3_O_4_ nanoparticles. Environ. Technol. Innov..

[41] Narwade M, Shaikh A, Gajbhiye KR, Kesharwani P, Gajbhiye V (2023). Advanced cancer targeting using aptamer functionalized nanocarriers for site-specific cargo delivery. Biomater. Res..

[42] Fallahizadeh S (2023). Enhanced photocatalytic degradation of amoxicillin using a spinning disc photocatalytic reactor (SDPR) with a novel Fe_3_O_4_@ void@ CuO/ZnO yolk-shell thin film nanostructure. Sci. Rep..

[43] Ghorbani M, Roshangar L, Rad JS (2020). Development of reinforced chitosan/pectin scaffold by using the cellulose nanocrystals as nanofillers: An injectable hydrogel for tissue engineering. Eur. Polymer J..

[44] Sun L (2017). Preparation and characterization of chitosan film incorporated with thinned young apple polyphenols as an active packaging material. Carbohydr. Polymers.

[45] Yousefi M (2024). Photocatalytic degradation of ciprofloxacin using a novel carbohydrate-based nanocomposite from aqueous solutions. Chemosphere.

[46] Feng X (2014). Liquid-exfoliated MoS_2_ by chitosan and enhanced mechanical and thermal properties of chitosan/MoS_2_ composites. Compos. Sci. Technol..

[47] Rajasekar S, Martin EM, Kuppusamy S, Vetrivel C (2020). Chitosan coated molybdenum sulphide nanosheet incorporated with tantalum oxide nanomaterials for improving cancer photothermal therapy. Arab. J. Chem..

[48] Aziz SB, Abdullah OG, Hussein SA, Ahmed HM (2017). Effect of PVA blending on structural and ion transport properties of CS: AgNt-based polymer electrolyte membrane. Polymers.

[49] Kasirajan K, Balaji M, Nithya P, Sundrarajan M (2020). International journal of biological macromolecules synthesis of biogenic chitosan-functionalized 2D layered MoS_2_ hybrid nanocomposite and its performance in pharmaceutical applications: In-vitro antibacterial and anticancer activity. Int. J. Biol. Macromol..

[50] Chang Y-P, Ren C-L, Qu J-C, Chen X-G (2012). Preparation and characterization of Fe_3_O_4_/graphene nanocomposite and investigation of its adsorption performance for aniline and *p*-chloroaniline. Appl. Surf. Sci..

[51] Nasseh N, Arghavan FS, Daglioglu N, Asadi A (2021). Fabrication of novel magnetic CuS/Fe_3_O_4_/GO nanocomposite for organic pollutant degradation under visible light irradiation. Environ. Sci. Pollut. Res..

[52] Nithya R, Thirunavukkarasu A, Sathya AB, Sivashankar R (2021). Magnetic materials and magnetic separation of dyes from aqueous solutions: A review. Environ. Chem. Lett..

[53] Han C, Huang G, Zhu D, Hu K (2017). Facile synthesis of MoS_2_/Fe_3_O_4_ nanocomposite with excellent photo-Fenton-like catalytic performance. Mater. Chem. Phys..

[54] Li S (2023). Efficient and selective removal of Hg (II) from water using recyclable hierarchical MoS_2_/Fe_3_O_4_ nanocomposites. Water Res..

[55] Zhou Q, Wang Y, Xiao J, Fan H, Chen C (2019). Preparation and characterization of magnetic nanomaterial and its application for removal of polycyclic aromatic hydrocarbons. J. Hazard. Mater..

[56] Rani M, Shanker U (2020). Metal oxide-chitosan based nanocomposites for efficient degradation of carcinogenic PAHs. J. Environ. Chem. Eng..

[57] Boon YH, Zain NNM, Mohamad S, Osman H, Raoov M (2019). Magnetic poly (β-cyclodextrin-ionic liquid) nanocomposites for micro-solid phase extraction of selected polycyclic aromatic hydrocarbons in rice samples prior to GC-FID analysis. Food Chem..

[58] Heydari R, Khavarpour M (2018). Adsorption of malachite green from aqueous solution by nanozeolite clinoptilolite: Equilibrium, kinetic and thermodynamic studies. Int. J. Eng..

[59] Wan, S.* et al.* Rapid and highly selective removal of lead from water using graphene oxide-hydrated manganese oxide nanocomposites. *J. Hazard. Mater.* **314**, 32–40 10.1016/j.jhazmat.2016.04.014 (2016). 10.1016/j.jhazmat.2016.04.014 27107233

[60] Moharram, M. A. K., Tohami, K., El Hotaby, W. M. & Bakr, A. M. Graphene oxide porous crosslinked cellulose nanocomposite microspheres for lead removal: Kinetic study. *React. Funct. Polymers* **101**, 9–19 10.1016/j.reactfunctpolym.2016.02.001 (2016).

[61] Pourfadakari S (2021). Optimization of electro-kinetic process for remediation of soil contaminated with phenanthrene using response surface methodology. Environ. Sci. Pollut. Res..

[62] Abdel-Shafy HI, Mansour MS (2016). A review on polycyclic aromatic hydrocarbons: Source, environmental impact, effect on human health and remediation. Egypt. J. Petrol..

[63] Amirdivani S (2019). Polycyclic aromatic hydrocarbons in milk and dairy products. Int. J. Dairy Technol..

[64] Ncube S, Madikizela L, Cukrowska E, Chimuka L (2018). Recent advances in the adsorbents for isolation of polycyclic aromatic hydrocarbons (PAHs) from environmental sample solutions. TrAC Trends Anal. Chem..

[65] Xi Z, Chen B (2014). Removal of polycyclic aromatic hydrocarbons from aqueous solution by raw and modified plant residue materials as biosorbents. J. Environ. Sci..

[66] Yang X, Yin Y, Zong Y, Wan T, Liao X (2019). Magnetic nanocomposite as sorbent for magnetic solid phase extraction coupled with high performance liquid chromatography for determination of polycyclic aromatic hydrocarbons. Microchem. J..

[67] Wang J, Chen Z, Chen B (2014). Adsorption of polycyclic aromatic hydrocarbons by graphene and graphene oxide nanosheets. Environ. Sci. Technol..

[68] Zhao J, Wang Z, Zhao Q, Xing B (2014). Adsorption of phenanthrene on multilayer graphene as affected by surfactant and exfoliation. Environ. Sci. Technol..

[69] Gupta H (2015). Removal of phenanthrene from water using activated carbon developed from orange rind. Int. J. Sci. Res. Environ. Sci..

[70] Wei Z (2022). High-efficiency adsorption of phenanthrene by Fe_3_O4-SiO_2_-dimethoxydiphenylsilane nanocomposite: Experimental and theoretical study. J. Hazard. Mater..

[71] Apul OG, Wang Q, Zhou Y, Karanfil T (2013). Adsorption of aromatic organic contaminants by graphene nanosheets: Comparison with carbon nanotubes and activated carbon. Water Res..

[72] Kumar JA (2019). Enhanced PAHs removal using pyrolysis-assisted potassium hydroxide induced palm shell activated carbon: Batch and column investigation. J. Mol. Liq..

[73] Yakout S, Daifullah A, El-Reefy S (2013). Adsorption of naphthalene, phenanthrene and pyrene from aqueous solution using low-cost activated carbon derived from agricultural wastes. Adsorpt. Sci. Technol..

[74] Ma J, Zhu L (2006). Simultaneous sorption of phosphate and phenanthrene to inorgano–organo-bentonite from water. J. Hazard. Mater..

